# Engineering Regenerative Fibrin Scaffold from Balanced Protein-Concentrate Plasma: Structural and Biochemical Characterization

**DOI:** 10.3390/pharmaceutics17111432

**Published:** 2025-11-05

**Authors:** Diego Delgado, Jon Mercader-Ruiz, Daniel Marijuán-Pinel, Pello Sánchez, Renato Andrade, João Espregueira-Mendes, Llanos Zuloaga, Jorge Knörr, Mikel Sánchez

**Affiliations:** 1 Advanced Biological Therapy Unit, Hospital MIKS, 01010 Vitoria-Gasteiz, Spain; diego.delgado@hospitalmiks.com (D.D.); jon.mercader@hospitalmiks.com (J.M.-R.); daniel.marijuan@hospitalmiks.com (D.M.-P.); pello.sanchez@hospitalmiks.com (P.S.); 2 Arthroscopic Surgery Unit, Hospital MIKS, 01010 Vitoria-Gasteiz, Spain; llanos.zuloaga@hospitalmiks.com (L.Z.); gorka.knorr@hospitalmiks.com (J.K.); 3 Clínica Espregueira—FIFA Medical Centre of Excellence, 4350-415 Porto, Portugal; randrade@espregueira.com (R.A.); jem@espregueira.com (J.E.-M.); 4 Dom Henrique Research Centre, 4350-415 Porto, Portugal; 5 Porto Biomechanics Laboratory (LABIOMEP), Faculty of Sports, University of Porto, 4200-450 Porto, Portugal; 6 School of Medicine, University of Minho, 4710-057 Braga, Portugal; 7 ICVS/3B’s-PT Government Associate Laboratory, 4710-057 Braga, Portugal; 8 3B’s Research Group—Biomaterials, Biodegradables and Biomimetics, University of Minho, Headquarters of the European Institute of Excellence on Tissue Engineering and Regenerative Medicine, Barco, 4805-694 Guimarães, Portugal; 9 Orthopedic Institut de Recerca Sant Joan de Déu, Esplugues de Llobregat, 08950 Barcelona, Spain; 10 Department of Pediatric Orthopedic Surgery, HM Nens Children’s Hospital, 08009 Barcelona, Spain

**Keywords:** platelet-rich plasma, balanced protein-concentrated plasma, fibrin scaffold, fibrinogen, biomechanics, autologous, regenerative medicine

## Abstract

**Background:** This study evaluates the impact of fibrinogen enrichment on the structural, mechanical, and bioactive properties of fibrin scaffold derived from balanced protein-concentrate plasma (BPCP), an autologous platelet-rich plasma (PRP) formulation with elevated extraplatelet content. **Methods:** A novel high-fibrinogen BPCP (HF-BPCP) scaffold was produced by combining BPCP platelet lysate with a concentrated fibrinogen solution at a 1:1 ratio, yielding nearly four-fold physiological fibrinogen levels. Comparative analyses between HF-BPCP and standard BPCP included platelet and fibrinogen quantification, scanning electron microscopy (SEM), rheology, indentation, adhesion testing, coagulation kinetics, retraction assays, biodegradation profiling, and growth factor (GF) release kinetics. **Results:** HF-BPCP displayed significantly denser fibrin networks with thinner fibers, higher porosity, and markedly faster coagulation times compared to BPCP. Mechanically, HF-BPCP exhibited greater stiffness, higher energy dissipation, and more stable adhesion, while almost eliminating scaffold retraction at 24 h. Despite improved early handling and structural integrity, HF-BPCP degraded more rapidly in vitro under tissue plasminogen activator exposure. GF release analysis showed reduced early peaks of platelet-derived factors (TGF-β1, PDGF-AB, VEGF) but sustained release thereafter, while extraplatelet factors (IGF-1, HGF) exhibited similar profiles between scaffolds. **Conclusions:** These results indicate that fibrinogen enrichment synergizes with the elevated extraplatelet protein profile of BPCP to enhance scaffold mechanical stability, handling properties, and controlled GF delivery. HF-BPCP combines the adhesive, structural, and bioactive features of fibrin sealants with the regenerative potential of PRP, offering a fully autologous alternative for clinical applications requiring rapid coagulation, high mechanical support, and sustained GF availability. Further preclinical and clinical studies are needed to evaluate therapeutic efficacy in the regenerative medicine field.

## 1. Introduction

The field of regenerative medicine has emerged as a transformative approach aimed at restoring the structure and function of damaged tissues by enhancing the body’s natural repair mechanisms [1,2]. In recent years, biologically derived products have gained significant attention as therapeutic agents across multiple medical disciplines [3,4,5,6]. Platelet-rich plasma (PRP) is one of the most commonly utilized therapies. It is obtained through centrifugation of the patient’s own blood, allowing the separation and concentration of platelets, which are rich in growth factors (GFs) and released upon platelet activation [7,8,9,10], stimulating and accelerating tissue repair processes such as cell proliferation, migration, and differentiation [11,12]. PRP allows different types of formulations, including liquid form for injections and solid form consisting of fibrin-based scaffolds [13,14]. However, the coagulation process involved in PRP scaffold tends to be relatively slow, which makes them less suitable for applications that require rapid matrix formation [15]. In fact, the type of activation affects the coagulation time. This can be endogenous, resembling a more natural environment but slowing down the process, or exogenous with inducing factors such as CaCl_2_ or thrombin [16]. Moreover, PRP scaffold exhibit poor mechanical properties and frequently limited capacity to adhere effectively to tissue due to the effect of the clot retraction [17,18].

To overcome these challenges, recent research has focused on modifying the structural and biochemical properties of fibrin-based scaffold [19,20,21]. Platelet-rich fibrin (PRF) [22,23,24,25] is characterized by its solid or gel-like consistency and high fibrin content. Nevertheless, these scaffold also present certain challenges such as the mechanical properties [26,27] and the content of white blood cells embedded within the fibrin network [28,29,30,31,32].

Another key factor influencing scaffold performance is fibrinogen content. Several studies have demonstrated that elevating fibrinogen concentration significantly enhances the physical robustness of fibrin matrices [18,33,34,35,36]. In clinical practice, fibrin sealants such as Tisseel^®^ (70–110 mg mL^−1^ fibrinogen) are widely used to provide immediate hemostasis and tissue adhesion, effectively stopping bleeding, and forming a barrier that helps in the healing process [37]. Nevertheless, their lack of platelets and allogenic origin limit their capacity to actively promote regeneration [36,38,39,40].

The biochemical composition of the PRP used is also critically important. Further innovations have focused on expanding the molecular composition of platelet concentrates beyond platelet-derived factors [11,41,42,43]. Balanced protein-concentrate plasma (BPCP) is a formulation that preserves a high platelet content while simultaneously enhancing the concentration of circulating plasma proteins, such as hepatocyte GF (HGF), insulin-like GF (IGF-1), α_2_-macroglobulin and also fibrinogen [44]. Thus, fibrin scaffold from BPCP showed nearly double fibrinogen levels compared to standard PRP, resulting in an increased GF release and improved stability without losing biocompatibility [35].

With the aim of consolidating all the properties described above, this study introduces a new scaffold derived from BPCP and enriched with autologous fibrinogen (HF-BPCP) and compares it with the BPCP scaffold. The primary aim is to elucidate how supplementing fibrinogen impacts scaffold ultrastructure, biomechanics, degradation, and GF release kinetics on the BPCP formulation. We hypothesize that increasing fibrinogen within the scaffold will further optimize fiber architecture and mechanical stability of BPCP, enabling more prolonged GF retention and structural support and thus, enhancing the scaffold’s utility in tissue regeneration.

## 2. Materials and Methods

### 2.1. Sample Collection and Preparation

#### 2.1.1. Donors

Healthy donors were selected ranging in age from 18 to 65 years old for this study. Whole blood was collected into 9 mL tubes (Greiner Bio-one, Kremsmünste, Austria) for PRP obtention, and 3.5 mL tubes (Greiner Bio-one, Kremsmünste, Austria) for the measurement of baseline hematological parameters, both containing 3.8% (*w*/*v*) sodium citrate. All plasma samples were stored at −80 °C. This study was conducted in accordance with the Declaration of Helsinki and approved by the Institutional Ethics Committee of the OSI Araba UCA-15/EE/24/HGL (2024-023, 30 May 2024). Informed consent was obtained from all subjects involved in this study.

#### 2.1.2. Balanced Protein-Concentrate Plasma Preparation

BPCP was obtained following the method described by Mercader et al. [41]. Briefly, 9 mL of blood were centrifuged at 1200× *g* for 8 min at room temperature (RT) and the entire plasma layer (PLR) was collected, while red and white blood cells were discarded. PLR contained levels of platelets and plasma circulating molecules similar to blood basal levels. After that, PLR was put in contact with 0.125 g mL^−1^ of HEAA hydrogel for 5 min to allow the plasma’s water absorption. Then, hydrogel powder was discarded by putting the plasma in contact with a 100 µm filtration unit (Biologix, Shandong, Jinan) on top of a 50 mL falcon tube (Corning, Corning, NY, USA). BPCP was collected by 500× *g* centrifugation for 2 min at RT. Finally, BPCP activation was carried out by adding 10% CaCl_2_ (20 µL mL^−1^) (Galenica senese, Monteroni d’Arbia, Italy) and it was kept at 37 °C for the release of platelet content and scaffold formation, which served as a control in this study.

#### 2.1.3. High Fibrinogen BPCP Preparation

HF-BPCP was obtained by mixing BPCP platelet lysate with a concentrated fibrinogen solution. BPCP platelet lysate, which contains thrombin activity and both extraplatelet and platelet-derived GFs, was obtained as described above (see Section 2.1.2). Additionally, the obtained BPCP lysate was sterilized by filtration through a Minisart ® NML Plus 0.2 μm filter (Sartorius, Goettingen, Germany) before being mixed with the fibrinogen solution.

To obtain concentrated fibrinogen solution, first, whole blood was centrifuged at 1500× *g* for 15 min to obtain platelet-free plasma. Then, 96% ethanol pharma-grade (PanReac AppliChem, Castellar del Vallès, Spain) was added gently to the obtained plasma at 10% (*v*/*v*) and incubated on ice for 30 min to allow protein precipitation. Then, tubes were centrifuged at 580× *g* for 8 min and supernatant was carefully removed. Fibrinogen pellets were warmed in a 37 °C water bath until fully dissolved. A final volume of 1.5–2 mL of the fibrinogen concentrate was achieved. Finally, BPCP lysate and obtained concentrated fibrinogen solution were mixed in 1:1 ratio. When the two components were mixed, there occurred an interaction between the thrombin in the BPCP lysate and the fibrinogen concentrate, forming the final fibrin matrix. 

### 2.2. Platelet and Fibrinogen Level Measurement

Platelet and fibrinogen levels were measured in different plasma formulations (blood, BPCP and HF-BPCP). Platelet count was performed in blood and BPCP using a Mindray BC-20s hematology analyzer (Mindray, Shenzhen, China). Fibrinogen levels were measured in blood, BPCP and HF-BPCP by a coagulation analyzer (STA Compact Max, Stago, Asnières-sur-Seine, France). To analyze fibrinogen concentration in HF-BPCP, 1:5 dilution was previously made with distilled water.

### 2.3. Morphological Analysis

The ultrastructural characteristics of the BPCP and HF-BPCP scaffolds were examined by scanning electron microscopy (SEM) using a Hitachi S-4800 microscope (Hitachi, Tokyo, Japan). The analysis was performed by the SGIKER service at the University of the Basque Country (UPV/EHU). After fibrin clot formation, samples were rinsed with PBS (3813; Sigma–Aldrich, Darmstadt, Germany) for 30 min and fixed in 2% glutaraldehyde (1121790025; Sigma–Aldrich, Darmstadt, Germany) prepared in Sorensen’s Buffer (11682-10-4L; Quimigen, Alverca do Ribatejo, Portugal). Subsequent treatment with osmium tetroxide (OsO_4_) and critical point drying was performed by the SGIKER service. Fibrin fiber diameter and pore quantity were quantified using ImageJ/FIJI software v 1.51 W (National Institutes of Health, Bethesda, MD, USA), following a detailed protocol described from a previous study [45].

### 2.4. Biomechanical Behavior

#### 2.4.1. Coagulation Kinetics

Clot formation kinetics for BPCP and HF-BPCP were measured by turbidimetry using the TECAN Infinite 200 PRO plate reader (TECAN, Zurich, Switzerland). BPCP was previously activated with 10% CaCl_2_ and subsequently, 100 µL was added to a 96-well plate (3628; Sigma–Aldrich, Darmstadt, Germany). For HF-BPCP clot formation measurement, both BPCP platelet lysate and concentrated fibrinogen solution were mixed (1:1 ratio) directly in each well. Absorbance was measured at 450 nm every 2 min for 1 h to ensure clot formation. Technical triplicates were carried out.

#### 2.4.2. Mechanical Tests

Biomechanical properties of both scaffolds were carried out by measuring Young’s modulus, dissipated energy and adhesion capacity. Young’s modulus and dissipated energy of the BPCP and HF-BPCP scaffolds were measured by instrumented indentation tests using a calibrated spherical indenter with a 5 mm diameter. The indenter was applied to the sample surface at a constant speed of 50 µm·s^−1^, and both the applied load and corresponding penetration depth were continuously recorded. Indentation proceeded until a depth equivalent to 20% of the sample’s initial thickness was reached, after which unloading was initiated by reversing the movement. Tests were conducted directly within 24-well plates (3524; Sigma–Aldrich, Darmstadt, Germany), using scaffolds with a thickness of 2 mm. All clots were prepared into each well. Measurements were taken at three distinct locations per sample, and an empty well served as a blank control.

To assess the adhesiveness of both matrix types, two cylinders were covered with a polypropylene gauze and impregnated with 100 µL of each formulation. 100 µL of BPCP was previously activated with 10% CaCl_2_ and then added into the gauze. For HF-BPCP analysis, 50 µL of BPCP platelet lysate and 50 µL of concentrated fibrinogen solution were mixed and immediately put on the gauze. Then, the gauzes were pressed together with a force of 30 mN and allowed to coagulate fully. Following matrix formation, adhesion strength was evaluated by attempting to separate the two adhered gauzes at a constant speed of 3 mm min^−1^. The adhesion strength was calculated based on the contact area between the gauzes and the maximum force required to achieve separation. 

All tests were performed using a Zwick/Roell ZwickiLine Z1.0 uniaxial testing machine (Ulm, Germany), operated by the Research Management Service of the University of Navarra (UNAV, Donostia-San Sebastián, Spain). The load cell used was a Zwick/Roell Xforce P with a maximum load of 50 N.

#### 2.4.3. Rheological Profile: Amplitude Sweep Oscillatory Test

The viscoelastic properties of the BPCP and HF-BPCP scaffolds were evaluated using an MCR 301 rheometer (Anton Paar GmbH, Graz, Austria). The measurements were carried out by the Research Management Service of the University of Navarra (UNAV, Donostia-San Sebastián, Spain). Clots were prepared by activating 1 mL of each formulation in 24-well plates, resulting in a matrix with an 8 mm diameter. Rheological measurements were conducted at RT using an oscillatory frequency of 10 rad s^−1^ and a strain range from 0.1% to 100%, beginning with an initial normal force of 0 N. Sample thickness was set to 0.5 mm, and the strain sweep consisted of 25 points. Data analysis was based on the linear viscoelastic region (LVE), where variations in storage modulus (G′) and loss modulus (G″) remained below 5%, in accordance with ISO guidelines.

#### 2.4.4. Retraction Capacity

Retraction was determined using a gravimetric method. Final volume of 500 µL of each formulation was prepared in 3.5 mL tubes. Regarding BPCP, 500 µL were activated with 10% CaCl_2_. For HF-BPCP clot formation, 250 µL of BPCP platelet lysate and 250 µL of concentrated fibrinogen solution were mixed. Duplicates were carried out and incubated at 37 °C for 30 min to allow complete coagulation.

After clot formation, the scaffolds were weighed (t_0_), and then incubated in 1 mL PBS at 37 °C to allow any shrinkage to occur. After 1 h and again after 24 h thereafter, the samples were placed in fresh PBS and the clots were weighed. Moreover, weight measurement continued for two weeks to monitor the weight of the clot.

The retraction percentage was calculated by comparing the initial weight of the clot to its final weight after a specific period of time. This formula calculates the percentage of weight loss due to clot retraction, measuring the degree to which the clot has contracted over time:

$$Retraction\;\%=\left(\frac{\left(W_{0}-W\right)}{W_{0}}\right)\;\times 100$$

where *W*_0_ is the initial weight of the scaffold; *W* is the weight of the scaffold after a specific period of time.

### 2.5. Biodegradation

Fibrinolysis of the scaffolds exposed to tissue plasminogen activator (tPA) was evaluated over a 5-day period by monitoring mass loss. tPA facilitates the conversion of plasminogen into plasmin, the enzyme responsible for fibrin degradation in vivo. In this context, tPA was used to accelerate the biodegradation of the fibrin clots [46]. The BPCP and HF-BPCP scaffolds were weighed (t_0_) one hour after activation. Following this, the samples were incubated at 37 °C in PC supplemented with tPA (0.25 µg mL^−1^, Abcam, Cambridge, UK). Weight measurements were taken every 24 h, without refreshing the PC. Experiments were conducted using plasma from five different donors, and technical triplicates were performed for each condition.

### 2.6. Release Kinetics

BPCP and HF-BPCP formulations were placed into a 12-well plate (3513; Sigma–Aldrich, Darmstadt, Germany) and incubated at 37 °C for 30 min to allow coagulation. After clot formation, the excess supernatant was carefully removed, and 3 mL of Dulbecco’s Modified Eagle Medium (DMEM, Gibco-Invitrogen, Grand Island, NY, USA) was added to each well. The plates were incubated again at 37 °C. The culture medium was collected on days 0, 1, 3, 6 and 10 and replaced, then stored at −80 °C for subsequent analyses. Experiments were conducted using samples from three different donors.

#### Platelet and Extraplatelet GF Concentration Measurements by Enzyme-like Immunosorbent Assay (ELISA) and Total Protein Release Analysis

ELISAs were carried out to detect both platelet and extraplatelet GFs in the collected DMEM. Measurements of the following proteins were taken in accordance with the manufacturer’s instructions: VEGF (DVE00; Bio-techne, Minneapolis, MI, USA), Transforming GF beta 1 (TGF-β1) (DB100C; Bio-techne Minneapolis, MI, USA), IGF-1 (DG100B; Bio-techne Minneapolis, MI, USA), HGF (DHG00B; Bio-techne Minneapolis, MI, USA) and Platelet-derived GF (PDGF-AB) (DHD00C; Bio-Techne Minneapolis, MI, USA). All protein levels were measured by absorbance and the corresponding concentrations were calculated by means of calibration curves (4PL). The total protein level released into the DMEM was measured using a Cobas c 501 analyzer (Roche, Basel, Switzerland).

### 2.7. Statistical Analysis

The distribution of the data was assessed by Shapiro–Wilk’s normality test. The different variables are presented as the mean ± SD for parametric data. The statistical significance of the differences between two groups were determined with Student’s *t*-test. The difference among more than two groups was validated with one-way or two-way ANOVA followed by Tukey/Dunnett’s multiple comparison test. Nevertheless, for non-parametric data, Kruskal–Wallis test was performed. Data were considered statistically significant when *p* < 0.05. GraphPad Prism® version 9.5 (GraphPad Software, San Diego, CA, USA) was used for the statistical analyses.

## 3. Results

### 3.1. Platelet and Fibrinogen Levels

**Figure 1:**
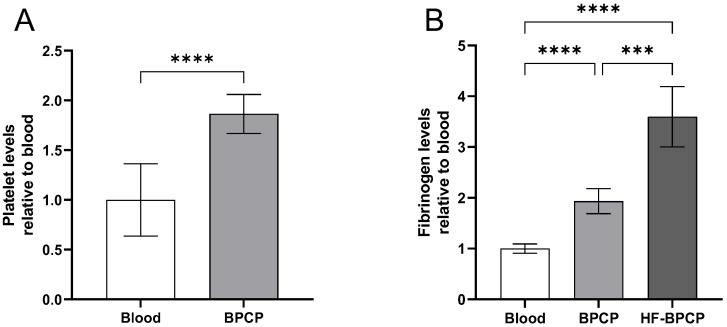
Platelet and fibrinogen levels in PRP formulations. Mean values of platelet in blood and BPCP are shown (**A**). Fibrinogen levels in blood, BPCP and HF-BPCP is represented (**B**). Error bars = standard deviation (*n* = 17 and 7, respectively). Statistically significant differences were calculated using paired t-Student test for the platelet count and Brown-Forsythe and Welch ANOVA tests for the fibrinogen levels (*** *p* < 0.001; **** *p* < 0.0001).

Platelet levels were measured in blood and BPCP to compare their biochemical composition (Figure 1A). Platelet analysis showed that the platelet content in BPCP (342.94 ± 117 × 10^3^ platelets µL^−1^) was twice compared to blood basal levels (186.18 ± 67.72 × 10^3^ platelets µL^−1^), and these differences were statistically significant (**** *p* < 0.0001). Platelet level was not measured in HF-BPCP, as fibrinogen concentrated was mixed with previously activated BPCP. Meanwhile, residual leukocytes and erythrocytes were below the detection limit. According to the Universal Coding System (UCS) for PRP studies described by Kon et al. [47], the products used in this study were 13-00-11.

Furthermore, fibrinogen content was measured both in blood, BPCP and HF-BPCP (Figure 1B). In whole blood, the fibrinogen concentration was measured at 3.7414 ± 0.3457 mg mL^−1^, consistent with established physiological reference ranges. BPCP exhibited a two-fold increase (7.1871 ± 0.5595 mg mL^−1^), being statistically significant compared to blood basal levels (**** *p* < 0.0001). Regarding HF-BPCP, as it was generated by mixing activated BPCP with a concentrated fibrinogen solution containing six times the fibrinogen concentration of whole blood (26.8929 ± 4.9550 mg mL^−1^), in a 1:1 volume ratio. As a result, fibrinogen concentration in HF-BPCP was half-reduced, showing an approximate two-fold increase in the fibrinogen content observed in BPCP (**** *p* < 0.0001).

### 3.2. Fiber Diameter and Pore Amount

**Figure 2:**
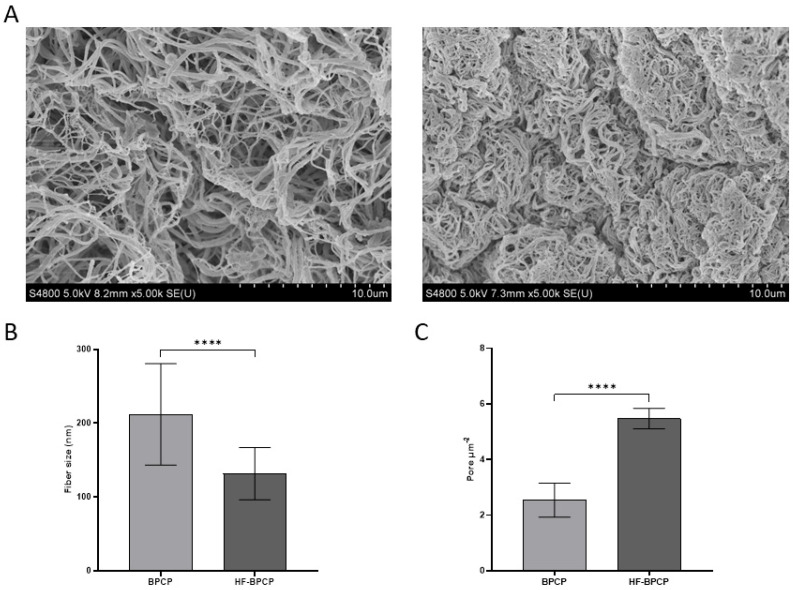
Morphological analysis of BPCP and HF-BPCP scaffolds. SEM images of BPCP (left) and HF-BPCP (right) (**A**). Scale bar 10 µm. Fiber diameter size in BPCP and HF-BPCP is expressed in nm (**B**). Porosity is represented by the number of pores per µm^2^. (**C**). Fiber diameter and number of pores were measured using the software ImageJ v 1.51W. Error bars = standard deviation (*n* = 4). Statistically significant differences were calculated using Student’s *t* test (**** *p* < 0.0001).

SEM analysis revealed significant statistical differences in fibrillar architecture between clots with varying fibrin content (Figure 2A). BPCP, with a lower fibrin concentration, exhibited thicker fibers (212 ± 68.72 nm) and a lower pore density (2.54 ± 0.61 pores µm^−2^). In contrast, HF-BPCP, with around twice the amount of fibrin, showed significantly thinner fibers (131.63 ± 35.31 nm) (**** *p* < 0.0001) and a higher pore density (5.47 ± 0.37 pores µm^−2^) (**** *p* < 0.0001) (Figure 2B,C).

### 3.3. Clot Formation Finetics Measured by Turbidimetric Absorbance

**Figure 3:**
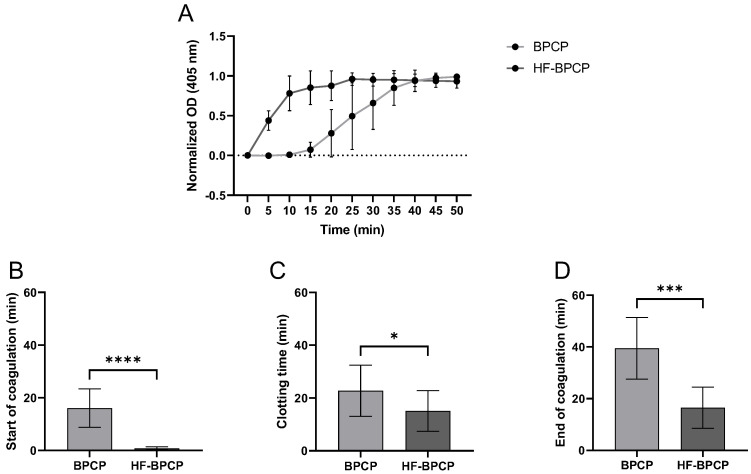
Coagulation kinetics of BPCP and HF-BPCP scaffolds. Normalized fibrin clotting kinetic curves over a 50 min period (**A**). The sigmoidal shape of the curves reflects the kinetics of fibrin polymerization. The duration of the different phases of the clotting process is shown in the figure: start of coagulation (**B**), clotting process (**C**) and end of coagulation (**D**). Time is expressed in minutes in all graphs. Error bars = standard deviation (*n* = 10). Statistically significant differences were calculated using Student’s *t* test (* *p* < 0.05; *** *p* < 0.001; **** *p* < 0.0001).

The kinetic coagulation pattern of both scaffolds was monitored by turbidimetry measuring absorbance at 405 nm over time (Figure 3A). The absorbance curves revealed that increasing fibrin concentration not only drastically shortens the initiation and polymerization time but also shifts the entire curve to the left, increasing the slope during the exponential phase. Indeed, BPCP exhibited a prolonged lag phase (16.1 ± 7.3 min). The growth phase proceeded at a moderately fast rate (22.75 ± 9.67 min) and reached completion around 40 min. HF-BPCP showed a very short lag phase (0.82 ± 0.6 min) and rapid coagulation (15.12 ± 7.72 min), achieving full stabilization by 16 min (Figure 3B–D). Thus, statistically significant differences were found between both scaffolds in all phases (**** *p* < 0.0001, * *p* = 0.035 and *** *p* = 0.0003, respectively).

### 3.4. Biomechanical Properties

Biomechanical and rheological properties of fibrin clots formed from both BPCP and HF-BPCP formulations were analyzed to assess how fibrinogen concentration influences the mechanical integrity and functional performance.

**Figure 4:**
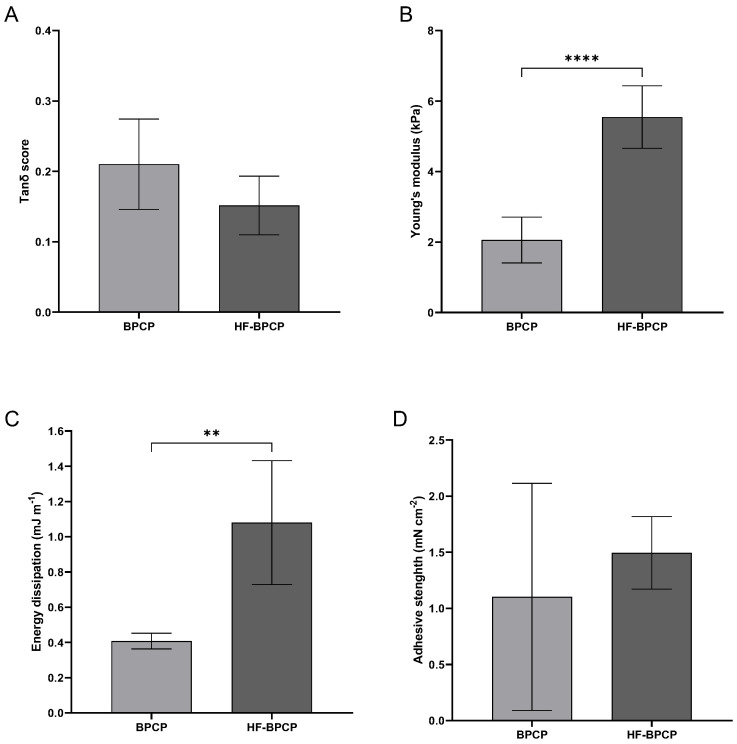
Mechanical properties of BPCP and HF-BPCP scaffolds. The graphs show the sweep amplitude data showing the tanδ score (viscoelasticity) (**A**), Young’s modulus (stiffness) (**B**), the dissipation energy (cushioning) (**C**) and the adhesion capacity (**D**) of the BPCP and HF-BPCP scaffolds. Error bars = standard deviation (*n* = 4–7). Statistically significant differences were calculated using Student’s *t* test (** *p* < 0.01; **** *p* < 0.0001).

Rheological measurements were conducted to determine the storage modulus (G′), loss modulus (G″), and the tan δ damping factor for each condition (Figure 4A). Both scaffolds exhibited tan δ values well below 1, indicative of predominantly elastic, solid-like behavior. Specifically, BPCP displayed a mean tan δ of 0.21 ± 0.06, while HF-BPCP yielded a slightly lower value of 0.15 ± 0.04. Despite the difference in fibrinogen content, no significant statistical difference was observed between the two groups (*p* > 0.05). 

The Young’s modulus was measured to assess the intrinsic stiffness of the fibrin networks under uniaxial deformation (Figure 4B). Interestingly, HF-BPCP exhibited a markedly higher modulus (5.55 ± 0.89 kPa) compared to BPCP (2.05 ± 0.65 kPa), being these difference statistically significant (**** *p* < 0.0001).

The capacity of the clots to dissipate mechanical energy during deformation was assessed as an indicator of their damping behavior and mechanical resilience (Figure 4C). This parameter reflects the potential of the matrix to buffer dynamic mechanical stresses, which is particularly relevant for in vivo applications subjected to repetitive mechanical forces. HF-BPCP showed a statistically significant higher energy dissipation (1.08 ± 0.35 mJ m^−1^) relative to BPCP (0.40 ± 0.04 mJ m^−1^) (** *p* = 0.0095), suggesting an enhanced ability to absorb and buffer mechanical stress. 

Lastly, adhesive capacity of the scaffolds was assessed, which is critical for their integration with surrounding tissues and their performance as biomaterials in regenerative therapies (Figure 4D). HF-BPCP again showed higher values (1.5 ± 0.32 mN cm^−2^) compared to BPCP (1.1 ± 1.01 mN cm^−2^). Nevertheless, BPCP showed high variability, indicating that BPCP has not consistent adhesion as the measurements were highly dispersed so that no statistically significant differences were found. 

### 3.5. Retraction Capacity

**Figure 5:**
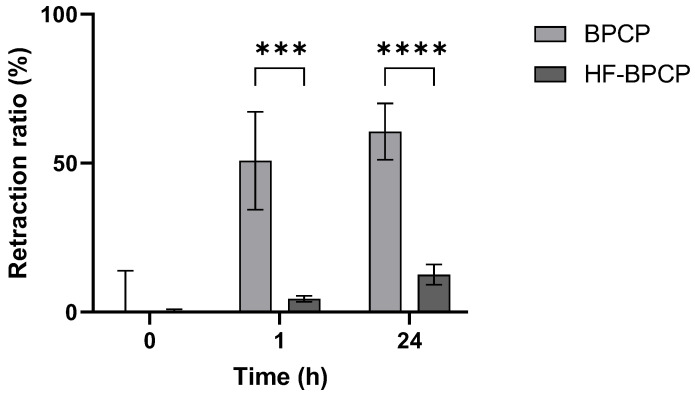
Retraction ratio of BPCP and HF-BPCP scaffolds. The retraction ratio (%) of BPCP and HF-BPCP scaffolds was measured up to 24 h. Error bars = standard deviation (*n* = 5). Statistically significant differences were calculated using Student’s *t* test (*** *p* < 0.001; **** *p* < 0.0001).

To assess the contractile behavior of the fibrin clots over time, their retraction capacity was evaluated by gravimetric analysis at two time points: 1 h and 24 h after clot formation (Figure 5).

BPCP scaffold showed a pronounced and rapid retraction profile, reaching 50.77 ± 16.39% volume reduction within the first hour and progressing to 60.55 ± 9.44% at 24 h. In contrast, HF-BPCP scaffold exhibited minimal contraction, with only 4.5 ± 0.99% retraction at 1 h and a modest increase to 12.5 ± 3.36% by 24 h. Statistical analysis revealed significant differences between the two scaffolds at both time points. The difference was already highly significant at 1 h (*** *p* = 0.000232), and became even more pronounced at 24 h (**** *p* = 0.000005). Moreover, no significant differences were seen between retraction % of BPCP scaffold at 1 h and 24 h (*p* = 0.2811). Nevertheless, registered retraction % at 24 of HF-BPCP scaffold was statistically higher than at 1 h (*** *p* = 0.0002) (Supplementary Material, Figure S1).

**Figure 6:**
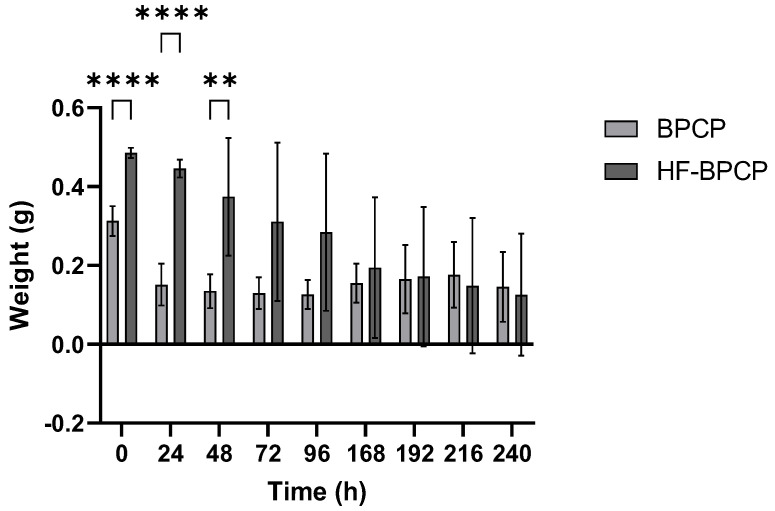
Scaffold weights obtained over time. The BPCP and HF-BPCP matrices were weighed every 24 h after incubation in fresh PBS over several days. Error bars = standard deviation (*n* = 11). Statistically significant differences were calculated using Student’s *t* test (** *p* < 0.01; *** *p* < 0.001; **** *p* < 0.0001).

Moreover, the absolute weight measurements were collected for two weeks to monitor the weight of the scaffolds. BPCP scaffold exhibited a sharp initial reduction in weight, from 0.31 ± 0.04 g at 0 h to 0.15 ± 0.05 g at 24 h. After this initial phase, the clot maintained a relatively stable weight throughout the full analysis period. In contrast, HF-BPCP scaffold began to show signs of degradation at 48 h. HFBPCP formulation showed a higher absolute weight compared to BPCP scaffold, being statistically significant upon 48 h (** *p* = 0.001). Nevertheless, HF-BPCP scaffold started to degradation at 72 h, as significant differences were found between weights recorded at t_0_ and that time point (* *p* = 0.0164) (Figure 6).

### 3.6. Biodegradation

**Figure 7:**
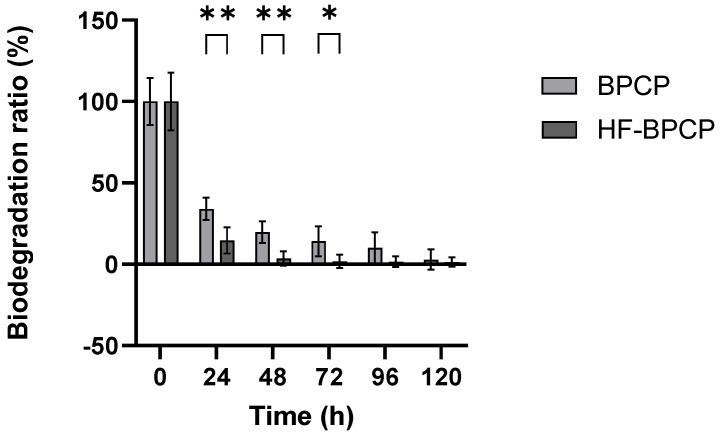
Biodegradation rates of BPCP and HF-BPCP scaffolds. Both scaffolds were exposed to 0.25 µg mL^−1^ tissue plasminogen-activator (tPA) for one week. Error bars = standard deviation (*n* = 5). Statistically significant differences were calculated using Student’s *t* test (* *p* < 0.05; ** *p* < 0.01).

The biodegradation profile of BPCP and HF-BPCP scaffolds were assessed by measuring the remaining mass every 24 h over a 5-day period (Figure 7). All values were normalized to the initial mass at t_0_ (100%).

The results showed that BPCP degraded progressively; 33.98% of the clot remained after 24 h, 19.94% after 48 h, 14.07% at 72 h, 10.1% at 96 h, and 2.79% at 120 h. HF-BPCP, despite having a higher fibrin concentration, degraded much more rapidly; only 14.5% remained after 24 h, 4% after 48 h, and it was fully degraded by 72 h. Moreover, significant differences in degradation rate between BPCP and HF-BPCP at 24, 48 and 72 h (*p* = 0.0079, *p* = 0.0079 and *p* = 0.0158, respectively) were observed. However, at 96 and 120 h, no significant differences were found (*p* = 0.127 and *p* < 0.99, respectively), as HF-BPCP had already been completely degraded by that point.

### 3.7. Release

**Figure 8:**
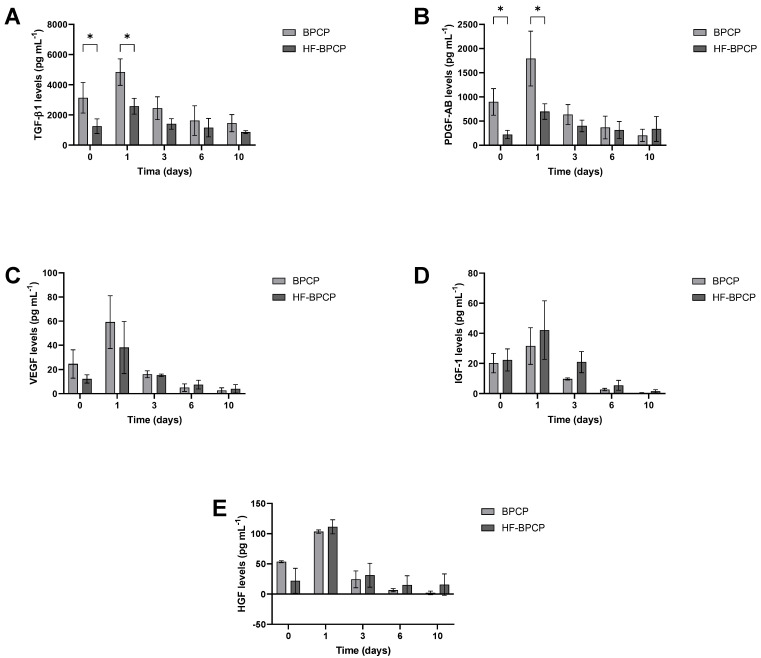
Release kinetics of both extraplatelet and platelet secreted GFs for a period of 10 days. Relative levels of TGF-1β (**A**), PDGF-AB (**B**), VEGF (**C**), IGF-1 (**D**), and HGF (**E**) released from BPCP and HF-BPCP scaffolds were measured by ELISA. Error bars = standard deviation (*n* = 3). Statistically significant differences were calculated using Student’s *t* test (* *p* < 0.05).

The capacity of BPCP and HF-BPCP scaffolds to release GF over time was measured by ELISA. Specifically, TGF-1β (Figure 8A) and PDGF-AB (Figure 8B), which are platelet-released GFs, VEGF (Figure 8C) and the extraplatelet GFs IGF-1 (Figure 8D), HGF (Figure 8E) were analyzed. Overall, a higher release was observed in both BPCP and HF-BPCP scaffolds at 24 h (Supplementary Material, Figures S2 and S3). Moreover, a similar pattern of release was observed in the case of extraplatelet factors as no significant differences were observed in IGF-1, HGF and VEGF levels. 

Regarding platelet secreted GFs, significant differences were observed at the two first time points. Indeed, TGF-1β and PDGF-AB release was higher in BPCP scaffold compared to HF-BPCP scaffold at 0 h (* *p* = 0.045 and * *p* = 0.019, respectively) and 24 h (* *p* = 0.016 and * *p* = 0.032, respectively). Nevertheless, although platelet derived levels were 2-fold reduced in HF-BPCP formulation as BPCP platelet lysate was diluted 1:2, no significant differences were observed in the release at days 3, 6, and 10, demonstrating a more sustained release over time.

### 3.8. Total Protein Release

**Figure 9:**
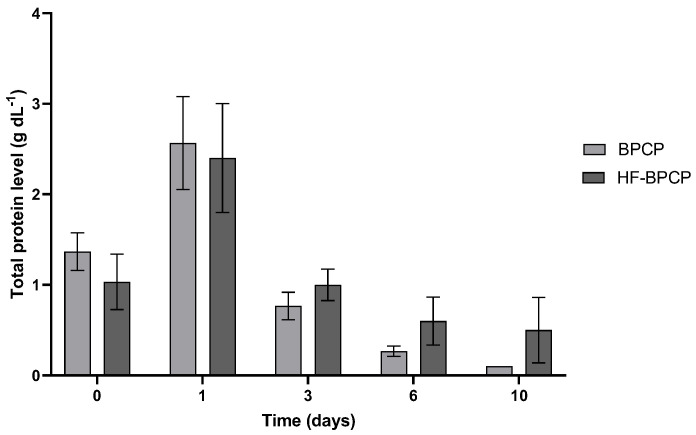
Total protein released from BPCP and HF-BPCP scaffolds. Total protein concentration (g dL^−1^) released from both plasma fibrin scaffolds over a 10-day period are shown. Error bars = standard deviation (*n* = 3). Statistically significant differences were calculated using Student’s *t* test.

In addition to GF levels, the total protein release content was analyzed in both scaffolds (Figure 9). Both BPCP and HF-BPCP exhibited same pattern of total protein release for 10 days. Nevertheless, a trend toward a more sustained total protein release was observed on days 3, 6, and 10 in the HF-BPCP scaffold compared to the BPCP scaffold.

## 4. Discussion

The main findings of this work showed that the combination of increased fibrinogen content and elevated extraplatelet factors in HF-BPCP offered features that could be beneficial for clinical use. Moreover, this new formulation integrated the key properties achieved by previous scaffolds, offering a comprehensive profile suitable for effective tissue regeneration. Key advantages included reduced retraction over time, enhanced mechanical properties, decreased coagulation time and similar GF release pattern compared to BPCP scaffold, being sustained over time. 

In the present study, HF-BPCP has been synthesized by mixing a concentrated fibrinogen solution and BPCP platelet lysate at a 1:1 ratio following the method described by Delgado D. et al. [34]. The BPCP platelet lysate provided the thrombin activity, necessary to initiate fibrin polymerization, resulting in the formation of a fibrin mesh. Moreover, it contained bioactive molecules such as GFs and cytokines that are known to support and enhance tissue regeneration [48]. This formulation contained nearly twice the fibrinogen concentration compared to BPCP and approximately four times the physiological levels found in peripheral blood. 

The two–fold increase in fibrinogen content in the HF-BPCP formulation compared to BPCP provided a greater substrate for fibrin polymerization, resulting in a denser and more protein-rich fibrin network [49,50]. This was demonstrated by microscopic analysis using SEM. The results revealed that the HF-BPCP scaffold exhibited a denser fibrin network characterized by thinner fibers and a higher number of pores per µm^2^ compared to the BPCP scaffold. These findings support that HF-BPCP formed a more compact scaffold and higher fibrinogen content decreased porosity of the scaffold [18,51,52].

Regarding clot density, the HF-BPCP scaffold demonstrated enhanced mechanical resistance, which is beneficial for supporting cellular microenvironments in bone, tendon, or cartilage defects and for maintaining local stability, especially in clinical scenarios where grafts or biomaterials must preserve their shape under physiological load [53,54,55]. Enhancement of biomechanical properties was evidenced by higher energy dissipation and an increased Young’s modulus, indicating enhanced cushioning capacity and stiffness [56]. Indeed, it has been reported that an inverse relationship has been observed between fiber diameter and stiffness, suggesting that larger fibrin fibers possess lower protein density and, consequently, reduced mechanical rigidity [57,58]. In line with this, Duong H. et al. [59] demonstrated that there was a corresponding increase in stiffness with increasing both thrombin and fibrinogen concentrations, being more notable for fibrinogen. Thus, the HF-BPCP scaffold demonstrated the ability to maintain its shape under tension, making it suitable for supporting tissues or anatomical spaces where structural integrity is required. Moreover, it exhibited enhanced capacity to absorb mechanical impacts and cyclic loading, which is particularly advantageous in dynamic tissues such as joints, tendons or muscles [60,61]. These results were consistent with the slight decrease in tan δ, indicating that the scaffold behaved more like an elastic solid, exhibiting increased resistance to permanent deformation and enhanced ability to recover its shape after deformation [62].

Furthermore, the observed increased mass in HF-BPCP scaffold correlated with greater volume and filling capacity, facilitating improved contact with the surrounding tissue margins and enhancing three-dimensional defect coverage, which is critical for guided tissue regeneration [63,64,65]. Scaffold retraction can also be a limiting factor when using them as fillers in surgical procedures, complicating handling and placement of fibrin scaffold [34]. It is well known that platelets play a central role in clot retraction, primarily mediated through the α_2_bβ_3_ integrin receptor abundantly expressed on their surface [66,67]. Activation of this receptor triggers a cascade of intracellular signaling events that induce cytoskeletal remodeling, ultimately resulting in clot compaction [68]. Moreover, Wufsus A. et al. [52] described that platelet induced fibrin scaffold retraction is attenuated by increasing fibrin and reducing platelet density. In the present study, HF-BPCP was formulated with those properties by using the fibrinogen concentrate and BPCP platelet lysate, thereby increasing fibrinogen content and removing the activated platelets from the formulation. So that, HF-BPCP scaffold showed with almost no retraction compared to BPCP scaffold.

Concerning coagulation time, the reduced coagulation rate of HF-BPCP formulation enables practical handling in the surgical setting, enabling appropriate preparation and application without procedural delays. This feature enhances intraoperative efficiency and is particularly advantageous in surgeries requiring rapid stabilization of the biomaterial at the defect site [35,69]. The increase in fibrinogen could also lead to a higher stability and adhesion capacity as reported by previous studies [70,71,72]. Furthermore, platelet content can influence matrix adhesion; for instance, matrices prepared from a reduced platelet content, exhibited superior adhesion compared to those derived from PRP [73]. An increased adhesion capacity may provide an advantage when using the scaffold for tissue treatment.

In addition to offering enhanced mechanical stability and handling performance, for wound treatment, the scaffold must also enable a sustained and controlled release of GFs and reparative molecules. In the present study, the release profiles of GFs from BPCP and HF-BPCP scaffolds revealed distinct patterns based on their origin and interaction with the fibrin matrix. The increase in fibrinogen content simultaneously led to a two-fold reduction in both platelet-derived and extraplatelet factors due to the 1:2 dilution applied during the preparation of the HF-BPCP formulation. As expected, the concentrations of released platelet-derived factors such as TGF-β1 and PDGF-AB were significantly reduced in HF-BPCP, which is consistent with the fact that this formulation contained half the platelet content compared to BPCP. Nevertheless, their release remained stable over the subsequent timepoints. It likely contributed to a more controlled and sustained release, minimizing the risk of excessive early stimulation and the disruption of the balance of cellular signaling pathways that could otherwise lead to undesirable inflammatory or fibrotic responses [74,75,76,77,78]. In contrast, the HF-BPCP scaffold moderated these initial release peaks, supporting a controlled and physiological delivery profile that closely replicates the dynamics of natural tissue repair. Regarding the extraplatelet factors, IGF-1 and HGF followed a similar release pattern to that of the BPCP scaffold. Thus, the temporal release profile of GFs appeared consistent, showing similar kinetics in both BPCP and HF-BPCP scaffolds. This suggests an efficient release of these factors from the HF-BPCP scaffold, potentially due to altered interactions with the denser fibrin network or reduced retention within the scaffold [79]. Indeed, the structural organization of fibrin fibers leading to smaller pores may contribute to sustained release profile over time. Regarding total protein release, both scaffolds exhibited a similar overall release pattern. However, the increased fibrinogen content in HF-BPCP led to a faster degradation rate, likely due to the greater fibrin exposure within its denser network, in addition to the thickness and highly crosslinked fibers [80]. Since fibrinogen constituted approximately 50% of the HF-BPCP scaffold, it is plausible that the majority of the proteins detected during the later stages of degradation correspond to fibrinogen breakdown products.

In addition to fibrinogen concentration, the activation method could have a direct impact on the PRP scaffold properties. Activation with CaCl_2_, as for BPCP, induces a gradual conversion of prothrombin to thrombin, promoting a decreased in polymerization rate and thicker fibrin fibers. In contrast, direct activation with thrombin and fibrinogen, as for HF-BPCP, leads to rapid fibrin formation, thinner fibers, and denser networks with distinct mechanical behavior and degradation kinetics. These activation-dependent differences have been shown to modulate fiber architecture, porosity, and lysis resistance of fibrin matrices [50,81,82].

This study presents several limitations that warrant consideration. The high volume of blood required for each assay restricts scalability and may introduce variability in the results, limiting the reproducibility and translational potential of the findings. Moreover, the lack of comparison with other fibrin-rich scaffolds such as commercial fibrin sealants, PRF products, or the HF-FM developed by Delgado et al. [34] prevents a clear assessment of the specific contribution of BPCP within the fibrin matrix. The absence of in vitro functional assays further constrains the interpretation of biological performance, as critical parameters such as cell adhesion, proliferation, and immunomodulatory activity remain uncharacterized. In particular, profiling the pro- and anti-inflammatory responses would strengthen understanding of the scaffold’s therapeutic relevance. Additionally, the rapid degradation of the HF-BPCP scaffold hampers long-term evaluation of properties such as swelling capacity and growth factor release, thereby limiting insights into sustained regenerative potential. Moreover, more comprehensive study is also required to elucidate the mechanisms underlying complex processes such as degradation, coagulation and growth factor release, which would provide a deeper understanding of these processes and inform strategies to optimize them. Finally, preclinical and clinical validation remains necessary to establish the scaffold’s biocompatibility, regenerative efficacy, and safety, which are essential for clinical translation.

## 5. Conclusions

HF-BPCP formulation showed a fibrinogen concentration nearly four times higher than baseline blood levels. This enrichment led to significantly improved mechanical properties, including enhanced stiffness and tensile strength, as well as faster coagulation kinetics and reduced scaffold contraction. Moreover, HF-BPCP scaffold demonstrated a more controlled and prolonged release of GFs, highlighting its potential as a robust and bioactive platform for regenerative applications requiring structural stability and sustained biological activity. However, further studies are required to assess its clinical applicability, including its biological effects and strategies to overcome its rapid in vivo degradation.

## Data Availability

The raw data supporting the conclusions of this article will be made available by the authors on request.

## Supplementary Materials

The following supporting information can be downloaded at: https://www.mdpi.com/article/10.3390/pharmaceutics17111432/s1, Figure S1: BPCP and HF-BPCP scaffold’s retraction capacity; Figure S2: Released GF levels form BPCP over time; Figure S3: Released GF levels form HF-BPCP over time.

## Abbreviations

The following abbreviations are used in this manuscript:

BPCP	balanced protein concentrate plasma
CaCl_2_	Calcium Chloride
ELISA	Enzyme-linked immunosorbent assay
GF	Growth factor
HEAA	Hydroxyethyl acrylamide
HF-BPCP	High fibrinogen BPCP
HGF	Hepatocyte growth factor
IGF-1	Insulin-like growth factor 1
LVE	Linear viscoelasticity
OsO4	Osmium tetroxide
PDGF-AB	Platelet-derived growth factor AB
PLR	Plasma layer
PRF	Platelet-rich fibrin
PRP	Platelet-rich plasma
RT	Room temperature
SEM	Scanning electron microscope
TGF-β1	Transforming growth factor beta 1
tPA	Tissue plasminogen activator
VEGF	Vascular endothelial growth factor
