# Supplementary material for: Engineering Regenerative Fibrin Scaffold from Balanced Protein-Concentrate Plasma: Structural and Biochemical Characterization
Source: Pharmaceutics. 2025 Nov 5;17(11):1432. doi: 10.3390/pharmaceutics17111432 (PMC12655132; doi:10.3390/pharmaceutics17111432)
Supplement: Supplementary file 1 [file pharmaceutics-17-01432-s001.zip › pharmaceutics-3945598-supplementary.pdf]

Supplementary Material

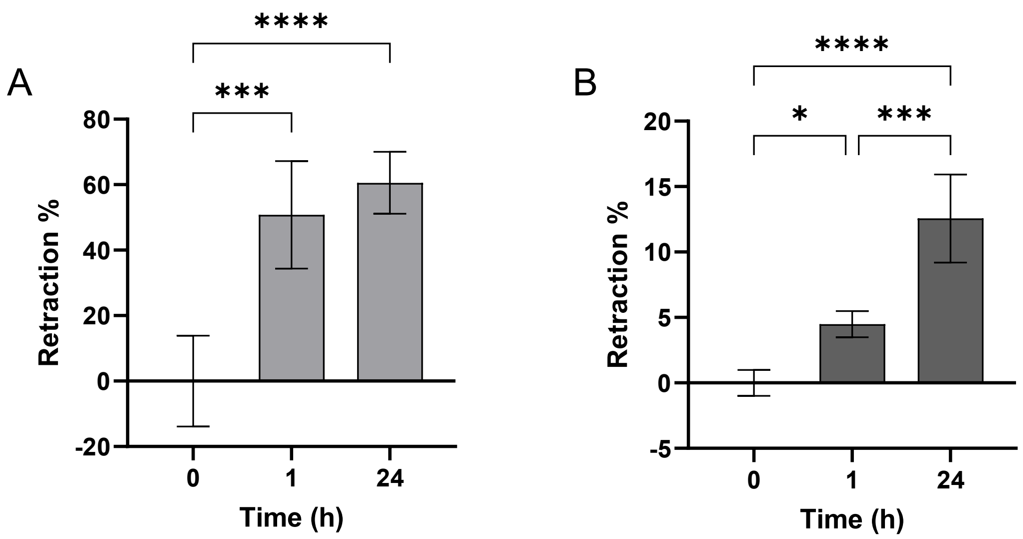

Figure S1: BPCP and HF-BPCP scaffold's retraction capacity. Scaffolds of 500 µL of BPCP (**A**) or HF-BPCP (**B**) were made and incubated in 1 mL of PBS at 37ºC for 1 and 24 h. Error bars = standard deviation (*n* = 11). Statistically significant differences were calculated using Student's t-test (** $p < 0.01$; *** $p < 0.001$; **** $p < 0.0001$).

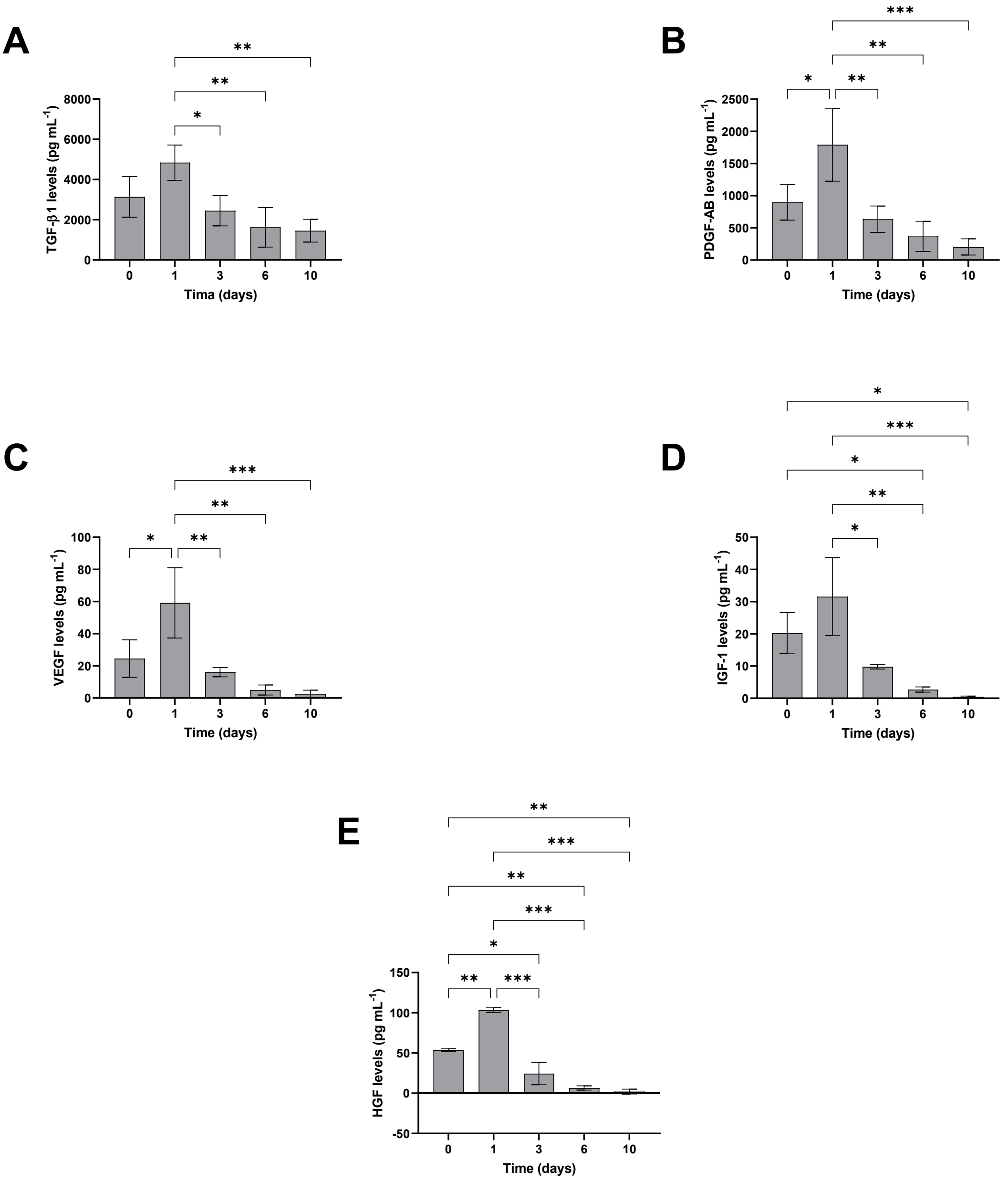

Figure S2: Released GF levels form BPCP over time. Concentration of TGF-β1 (**A**), PDGF-AB (**B**), VEGF (**C**), IGF-1 (**D**) and HGF (**E**) from BPCP scaffold for 10 days. Error bars = standard deviation (n = 3). Statistically significant differences were calculated using Ordinary ANOVA one-way analysis (* $p< 0.05$, ** $p < 0.01$; *** $p < 0.001$; **** $p < 0.0001$).

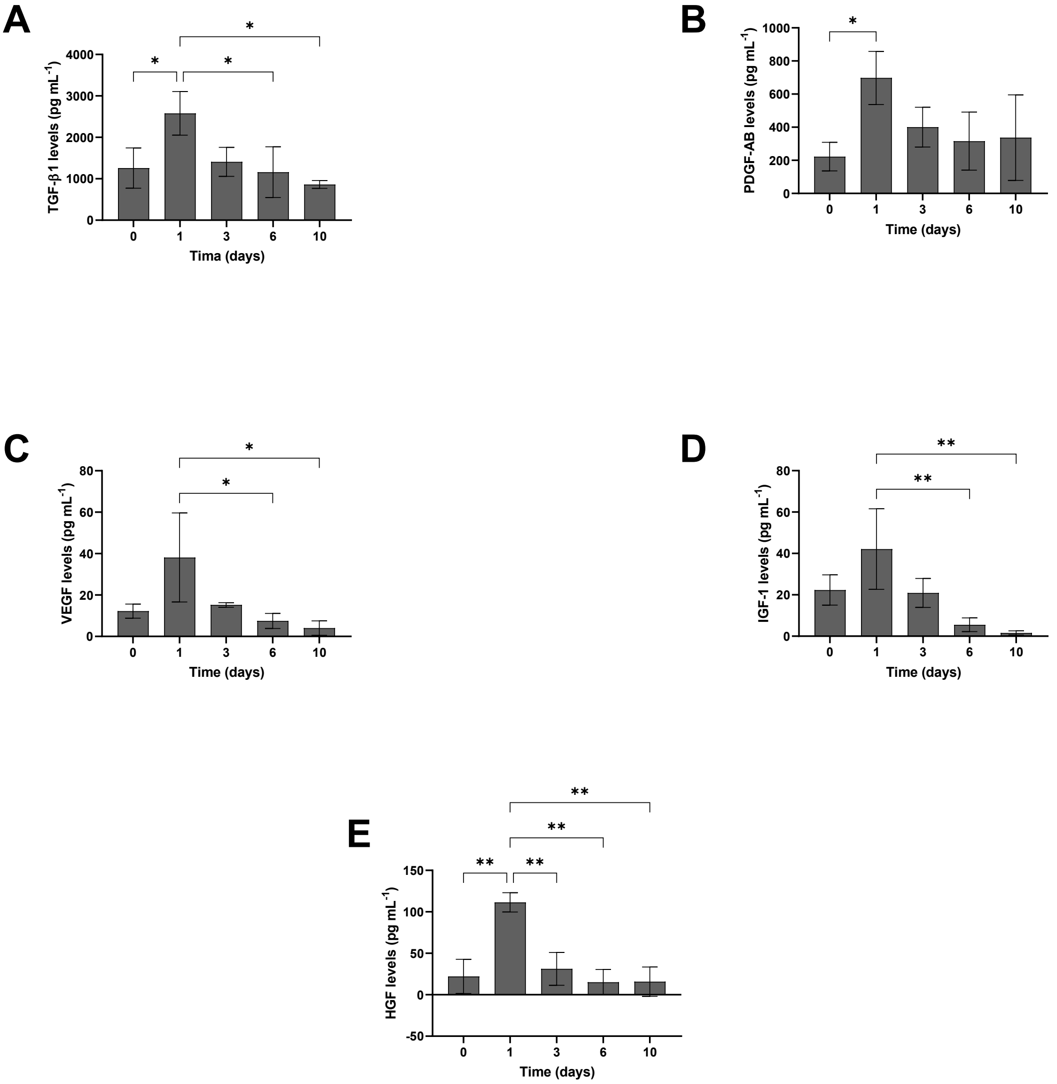

Figure S3: Released GF levels form HF-BPCP over time. Concentration of TGF-β1 (**A**), PDGF-AB (**B**), VEGF (**C**), IGF-1 (**D**) and HGF (**E**) from BPCP scaffold for 10 days. Error bars = standard deviation (n = 3). Statistically significant differences were calculated using Ordinary ANOVA one-way analysis (* $p < 0.05$, ** $p < 0.01$; *** $p < 0.001$; **** $p < 0.0001$).
